# Cross-reactivities in conjugation reactions involving iron oxide nanoparticles

**DOI:** 10.3762/bjnano.16.106

**Published:** 2025-08-29

**Authors:** Shoronia N Cross, Katalin V Korpany, Hanine Zakaria, Amy Szuchmacher Blum

**Affiliations:** 1 Department of Chemistry, McGill University, 801 Sherbrooke Street West, Montreal, QC H3A 0B8, Canada

**Keywords:** click chemistry, copper-catalyzed azide–alkyne cycloaddition, disulfide reduction, iron oxide nanoparticles, thiol–maleimide Michael addition

## Abstract

The preparation of multimodal nanoparticles by capping magnetic iron oxide nanoparticles (IONPs) with functional organic molecules is a major area of research for biomedical applications. Conjugation reactions, such as carbodiimide coupling and the highly selective class of reactions known as “click chemistry”, have been instrumental in tailoring the ligand layers of IONPs to produce functional biomedical nanomaterials. However, few studies report the controls performed to determine if the loading of molecules onto IONPs is due to the proposed coupling reaction(s) employed, or some other unknown interaction with the IONP surface. Herein, we use 3,4-dihydroxybenzoic acid-functionalized IONPs (IONP-3,4-DHBA) as a platform upon which carbodiimide coupling can be used to conjugate clickable small molecules for further functionalization using two common click reactions, namely, the copper-catalyzed azide–alkyne cycloaddition (CuAAC), and the thiol–maleimide Michael addition reactions. Through the judicious use of controls, we demonstrate significant cross-reactivities of amines, thiols, maleimides, and common disulfide reducing agents with surface Fe of IONPs and show how these unwanted interactions can produce false positive results. Without proper controls, these can lead to erroneous conclusions about the efficacy of conjugation reactions, which can have detrimental impacts on the functionality and safety of IONPs in biomedical applications.

## Introduction

Iron oxide nanoparticles (IONPs) have been the subject of an immense body of research in the field of biomedicine, where their magnetic properties are appealing for such applications as MRI contrast agents [1], tumor hyperthermia [2], and magnetic drug delivery [3]. Magnetite (Fe_3_O_4_) nanoparticles (NPs), in particular, have been explored for these applications, due to their low toxicity, biocompatibility, and high saturation magnetization [4]. These applications require reliable and controlled surface functionalization to impart desired functionality, such as tissue targeting and drug payload delivery. The ability to chemically modify the ligand layer of IONPs, while maintaining their morphology and magnetic properties, is thus paramount to the preparation of functional IONPs.

The use of highly selective conjugation reactions such as 1-ethyl-3-(3-dimethylaminopropyl)carbodiimide (EDC) coupling [5] and “click” chemistry reactions [6] have proven to be effective tools in the synthesis of novel functional materials. Carbodiimide coupling is an invaluable tool for the coupling of carboxylic acids to amines, through amide bond formation. The combination of EDC and *N*-hydroxysuccinimide (NHS) to activate carboxylates produces NHS ester intermediates, which are excellent leaving groups and are also more stable than the *O*-acylisourea intermediates of the EDC-only activation [5]. The use of EDC and EDC/NHS coupling has been demonstrated in the literature to prepare functionalized IONPs, using either amine- or carboxylic acid-functionalized IONPs, especially when biomolecules, such as proteins or peptides, are involved [3,7–14].

Sharpless et al. [6] have generally defined “click chemistry” as chemical processes that are highly selective, have a high thermodynamic driving force, use simple reaction conditions, produce no interfering byproducts, produce products that are stable under physiological conditions, and can be purified easily using non-chromatographic means. The copper-catalyzed azide–alkyne cycloaddition (CuAAC) reaction is one of the most commonly used click reactions and shows high specificity [15–16]. The Cu(I) catalyst increases the coupling rate of azides and alkynes by up to seven orders of magnitude, forming triazole rings, which possess exceptional chemical stability [16]. For these reasons, the CuAAC has been utilized extensively for the preparation of functional IONPs capped with either alkynes or azides [8,13,17–27].

The thiol–maleimide Michael addition reaction is another commonly employed click reaction [28–31], which can be performed at room temperature by simply mixing thiol- and maleimide-functionalized components, with no other additions required. The thiol addition to the maleimide double bond is highly favorable due to the electron withdrawing properties of its carbonyls, as well as the release of ring strain accompanying the formation of the thioether linkage [29]. Indeed, the versatility of this coupling reaction has led to numerous reports of its use with IONPs, through surface functionalization with either thiols or maleimides [10,32–39]. The IONP surface can also be capped with disulfide ligands, such as glutathione disulfide (GSSG), followed by cleavage of the disulfide bonds with reducing agents to produce free thiols (GSH) for maleimide coupling [40–41].

In the field of IONP functionalization, commonly used coupling reagents are generally assumed to be orthogonal, and little focus is placed on their potential cross-reactivities. It is also assumed that the multifunctional ligands used for the stabilization of the IONPs themselves bind with a single binding mode, which may be an incorrect assumption that can impact downstream functionalization [42]. For the purposes of multifunctional IONPs, which can possess complex, modular surface chemistries, these considerations are especially important. The reactivity of the iron oxide surface itself is often neglected, but it should be noted that the use of thiols [43–46] and amines [47–48] as anchoring groups for IONP ligands has been reported in the literature, so it is clear that a binding affinity exists between these groups and surface Fe. From this standpoint, it is evident that proper controls are necessary to determine the cross-reactivity of these groups with IONPs.

**Figure 1:**
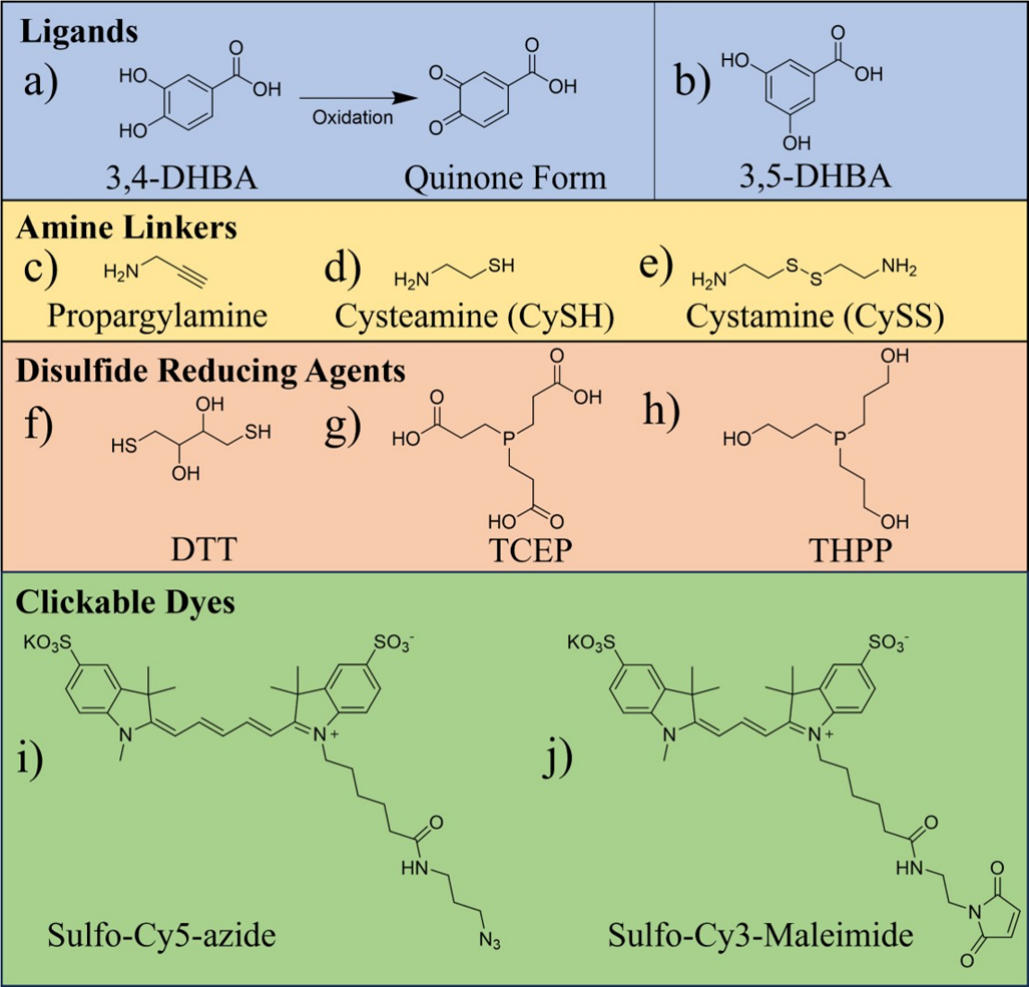
Structural formulae of ligands, amine linkers, disulfide reducing agents, and clickable dyes used in this study.

Herein, we report on the cross-reactivities of amines, thiols, maleimides, and common disulfide reducing agents with the IONP surface, and how these unwanted interactions can influence the interpretation of conjugation results. We use IONPs capped with 3,4-dihydroxybenzoic acid (IONP-3,4-DHBA) as a platform upon which we can perform common conjugation reactions. 3,4-DHBA (Figure 1a) is a catechol derivative, which bears a carboxylic acid group, capable of ionizing in solution (p*K*_a_ = 4.46) [49]. The normal binding mode of 3,4-DHBA to the IONP surface is through its catechol group [42], which has an exceptionally high affinity for Fe^3+^ [48], leaving its carboxylic acid group solution-exposed. We take advantage of the solution-exposed carboxylate group of 3,4-DHBA to produce IONPs that are amenable to further functionalization using EDC coupling, without sacrificing the small particle size, which is critical for maintaining their magnetic properties [50] and increasing their half-life in the bloodstream [51–52]. In the context of magnetic resonance imaging, small molecule ligands also offer advantages over thicker, polymeric coatings as superior contrast agents [53–55]. To investigate amine cross-reactivities, we use propargylamine (PPA, Figure 1c) and a series of primary amine-containing dyes. Cysteamine (CySH, Figure 1d) is used to assess thiol cross-reactivities, while cystamine (CySS, Figure 1e) is used as a ligand to investigate cross-reactivities of the disulfide reducing agents, dithiothreitol (DTT, Figure 1f), tris(2-carboxyethyl)phosphine (TCEP, Figure 1g), and tris(3-hydroxypropyl)phosphine (THPP, Figure 1h). We probe cross-reactivities in the CuAAC and thiol–maleimide Michael addition reactions using two dyes, Cy5-azide (Figure 1i) and Cy3-maleimide (Figure 1j), respectively. Oxidation of 3,4-DHBA forms an *o*-quinone (Figure 1a), which has a greatly diminished affinity for the IONP surface [48], but can still bind through its carboxylate group. To rule out the possibility of cross-reactivity with quinones, in the case of 3,4-DHBA, we incorporate a set of controls using 3,5-DHBA ligands (Figure 1b), which cannot form stable quinones due to their *meta* hydroxy groups [56]. Few studies report the controls performed to determine if the loading of functional molecules onto IONPs is due to the proposed coupling reaction(s) employed, or some other unknown interaction with the IONP surface. We demonstrate here that a failure to account for these cross-reactivities can lead to erroneous conclusions about the efficacy of common conjugation reactions on the IONP surface. This, in turn, can have detrimental impacts on the functionality and safety of IONPs in biomedical applications.

## Results and Discussion

### Use of propargylamine for EDC coupling to IONPs and CuAAC coupling of Cy5-azide

**Figure 2:**
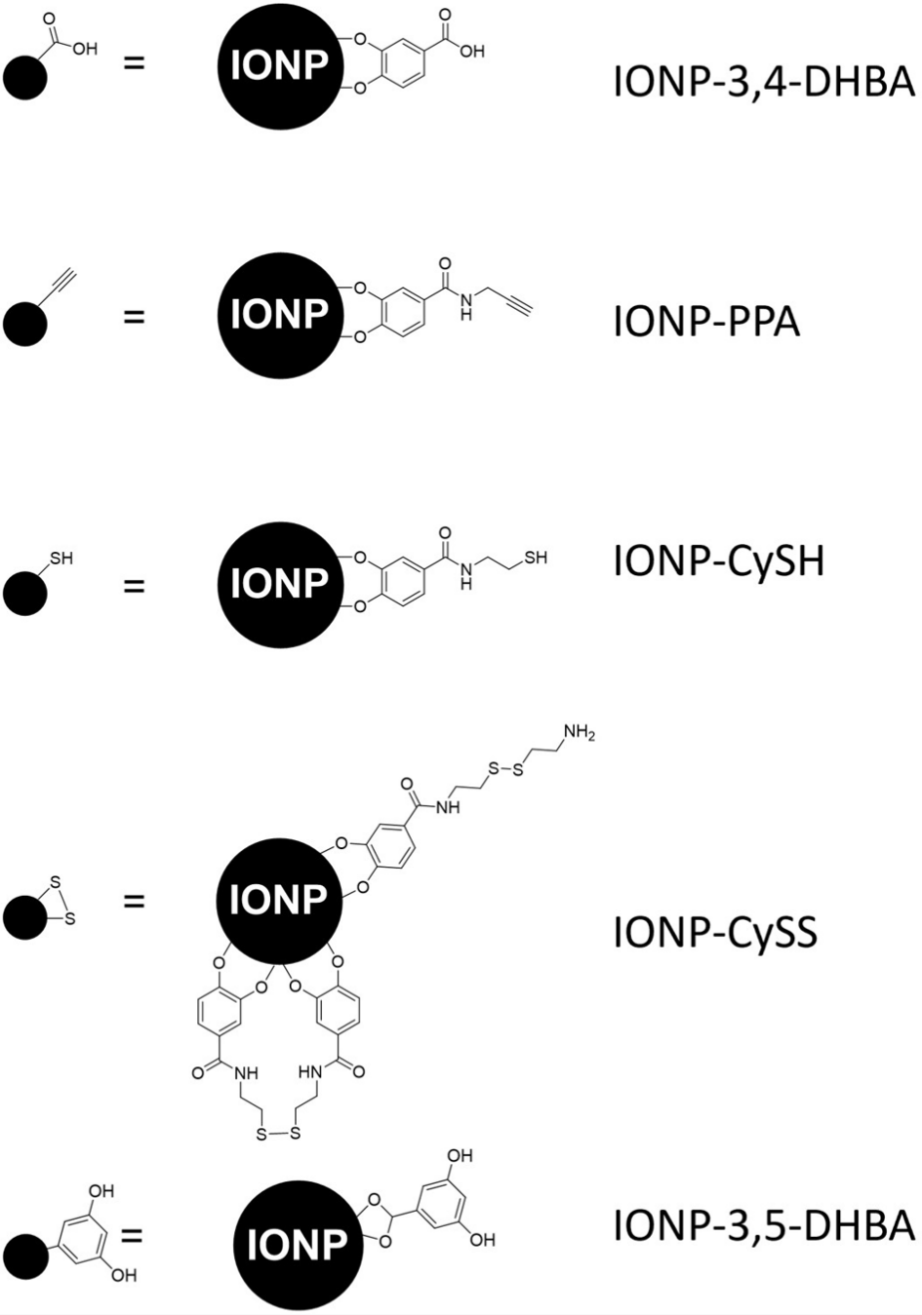
Legend of labeling schemes used within the figures throughout the text. Left column: the black circles represent IONPs (not to scale), while only the relevant functional groups of the ligands are illustrated for clarity, as used throughout the text. Right column: IONPs (not to scale) with the full structural formulae of the attached ligands illustrated.

**Figure 3:**
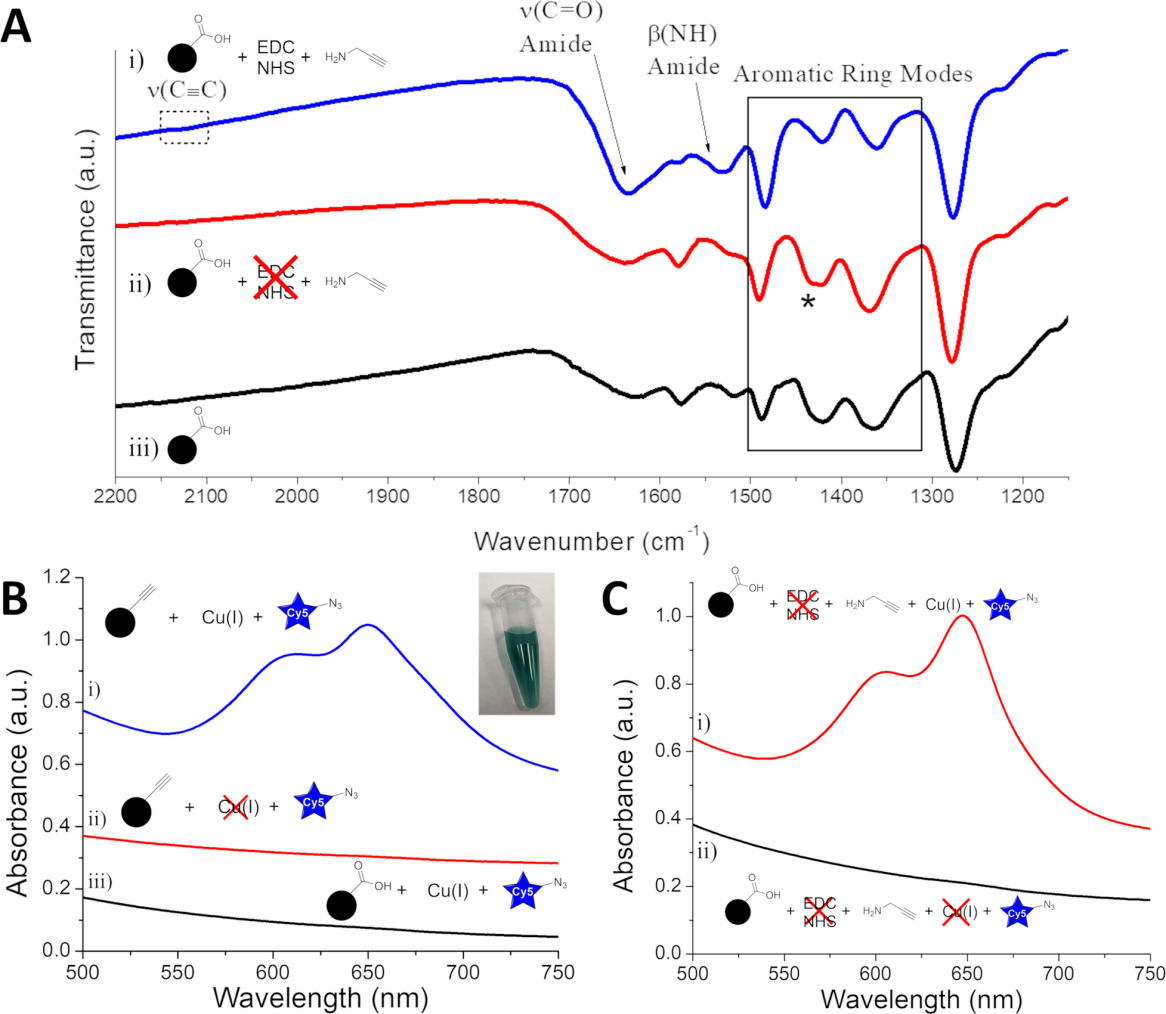
FTIR and UV–vis characterization of IONP-PPA and controls. (A) FTIR spectra of i) IONP-PPA, (ii) EDC-free PPA control, and iii) IONP-3,4-DHBA. (B) UV–vis spectra of i) IONP-PPA CuAAC product, ii) Cu-free CuAAC control, and iii) alkyne-free CuAAC control. Inset: photograph of an aqueous suspension of IONP-PPA CuAAC product. (C) UV–vis spectra of the CuAAC products of i) EDC-free PPA control, and ii) EDC-free PPA control without Cu catalyst.

PPA is a commonly used small molecule linker, which allows for the carbodiimide coupling of its amine group to a carboxylate group on one end and simultaneous coupling of its alkyne group to an azide, through the CuAAC, on the other end. We first activated the carboxylate groups of IONP-3,4-DHBA with EDC and NHS at pH 5 in MES buffer, to form the amine-reactive NHS ester, which could then be coupled to PPA at pH 7.8, in MOPS buffer. For clarity, simplified schemes are used to label spectra of different IONP preparations throughout this text, in which IONPs are represented by black spheres, the bound ligands are not drawn to scale, and certain sections of ligand molecules may be omitted. Figure 2 provides a legend with the more detailed structural formulae of the IONP surfaces for each preparation. FTIR analysis of IONP-PPA shows a very weak bump in the spectrum at 2122 cm^−1^, which may be attributed to the alkyne group (Figure 3A.i, dashed box), however, the inherently weak intensity of the alkyne ν(C≡C) band makes this assignment difficult [57]. In this same curve, we can observe the appearance of a ν(C=O) band at 1634 cm^−1^ and a δ(NH) band at 1530 cm^−1^, which together are consistent with the presence of amides [57]. While FTIR provides some evidence of PPA coupling to the IONP surface, it is not conclusive; thus, we turned to an indirect means of detection by utilizing the CuAAC coupling reaction to bind Cy5-azide dye to the alkyne groups on the IONP surface, if present. The dye can then be detected using UV–vis spectroscopy.

Following the CuAAC reaction with Cy5-azide, we immediately see a visible change in the color of the IONPs from brown to deep blue-green (Figure 3B, inset). The UV–vis spectrum of the IONP-PPA CuAAC product (Figure 3B.i) shows two broad peaks at ≈610 and 650 nm, which correspond to the pure Cy5 absorption bands (Supporting Information File 1, Figure S1.i); however, that alone does not prove the successful binding of PPA. For that purpose, we ran two controls, that is, one in which IONP-PPA was subjected to CuAAC reaction conditions, but without the addition of the Cu(I) catalyst (Figure 3B.ii), and one in which IONP-3,4-DHBA (no alkynes) was subjected to the full CuAAC reaction (Figure 3B.iii). We see that, in both controls, there is no detectable Cy5 loading on the IONPs, which suggests that the loading of Cy5-azide is, in fact, through the CuAAC, thus providing strong indirect evidence of the successful binding of PPA to the IONP surface. These results further demonstrate that there is no detectable cross-reactivity between Cy5-azide and the IONP surface.

We have demonstrated that PPA has been successfully loaded onto the IONP surface, producing alkyne-functionalized IONPs, which can be coupled to azides through the CuAAC, with no detectable cross-reactivity. At this point, it may be tempting to label the conjugation reaction as a success. However, an important step in the synthesis has been neglected: We have not provided evidence that the binding of PPA is through the intended pathway, that is, EDC/NHS coupling to the 3,4-DHBA carboxylate group. For this purpose, IONP-3,4-DHBA was first incubated in MES buffer (pH 5), in the absence of EDC and NHS, to mimic the activation conditions, and subsequently reacted with PPA in MOPS buffer at pH 7.8. Once again, the CuAAC with Cy5-azide was used to probe the product. Surprisingly, Figure 3C.i shows significant Cy5-azide loading in the absence of EDC/NHS activation, comparable to the EDC/NHS-activated product (Figure 3B.i), suggesting that the binding of PPA to IONP-3,4-DHBA is at least partially independent of EDC/NHS activation. In the absence of the Cu(I) catalyst, we see no trace of Cy5 on the EDC-free control (Figure 3C.ii), which confirms that Cy5-azide is loading to the control through the CuAAC. This further provides evidence that, in the absence of EDC/NHS activation, PPA must still somehow bind to IONP-3,4-DHBA through its amine group, leaving the alkyne solution-accessible.

The FTIR spectrum of the EDC/NHS-free PPA control (Figure 3A.ii) does not show the presence of the 2120 cm^−1^ alkyne ν(C≡C) band, which may suggest a lower PPA loading than IONP-PPA. We also do not observe the 1635 cm^−1^ amide ν(C=O) and 1530 cm^−1^ β(NH) bands in the control spectrum (Figure 3A.ii), as there should be no amide bond formation in the absence of EDC/NHS activation. We further note the absence of vibrational bands associated with primary ammonium salts (≈1500–1600 cm^−1^) [57], which might indicate the formation of a carboxylate–ammonium ionic bond.

For our purposes, it is important to acknowledge the reactivity of the 3,4-DHBA ligands we are using and their potential oxidation products towards common functional groups. The reactivity of quinones towards amines and thiols is well established, although thiols tend to react faster and form more stable products than amines [58]. The extent to which 3,4-DHBA ligands are oxidized to quinones (or semiquinones) on the IONP surface is not known, and FTIR analysis provides ambiguous evidence of their presence [42]; however, we must keep these possible reaction pathways in mind. If the PPA amines were reacting with 3,4-DHBA quinones on the IONP surface, this would result in the formation of tetra-substituted aromatic ring structures, derived from the tri-substituted DHBA ring (see Figure 9C.ii). Several aromatic ring bending and stretching modes are identified in the region of ≈1500–1330 cm^−1^, with the exception of the shoulder labeled (*) at 1432 cm^−1^, which is assigned to the ν(COO^−^)_s_ band [42]. The remaining peaks in this region show good qualitative agreement between the IONP-3,4-DHBA (Figure 3A.iii) and EDC-free control samples, which suggests similar tri-substituted ring structures, thereby providing evidence against the formation of tetra-substituted rings, making quinone reactions unlikely.

There is an important difference observed in the lineshapes of the post-CuAAC UV–vis spectra of IONP-PPA, and the EDC/NHS-free control (Figure 2B.i and 2C.i). Most notably, the control appears to be composed of relatively narrow lineshapes, while that of IONP-PPA appears noticeably broader. The relative intensities of the two Cy5 peaks are different in the two samples as well. When compared to the spectrum of pure Cy5-azide in solution (Supporting Information File 1, Figure S1.i), we see that both samples show significant broadening, and a change in the relative intensities of the two peaks. Similar broadening and lineshapes (relative intensities) were observed for Cy5-hexafluorophosphate thin films, and attributed to H-aggregate formation [59]. With the FTIR results in mind, we propose that the binding mode observed in the control sample involves only PPA bound directly to the IONP surface through Fe–amine coordination (Fe-PPA), and this configuration does not allow for sufficient coverage to promote extensive H-aggregate formation on the IONP surface. In the case of IONP-PPA, in addition to Fe-PPA binding, some of the PPA also couples to the NHS-activated 3,4-DHBA (3,4-DHBA-PPA), as intended, and as suggested by the above FTIR analysis. On the IONP-PPA surface, Cy5-azide can then bind to both the Fe-PPA, as well as the 3,4-DHBA-PPA, thereby producing a denser packing of Cy5, which allows for the formation of H-aggregates, resulting in the observed broadening.

**Figure 4:**
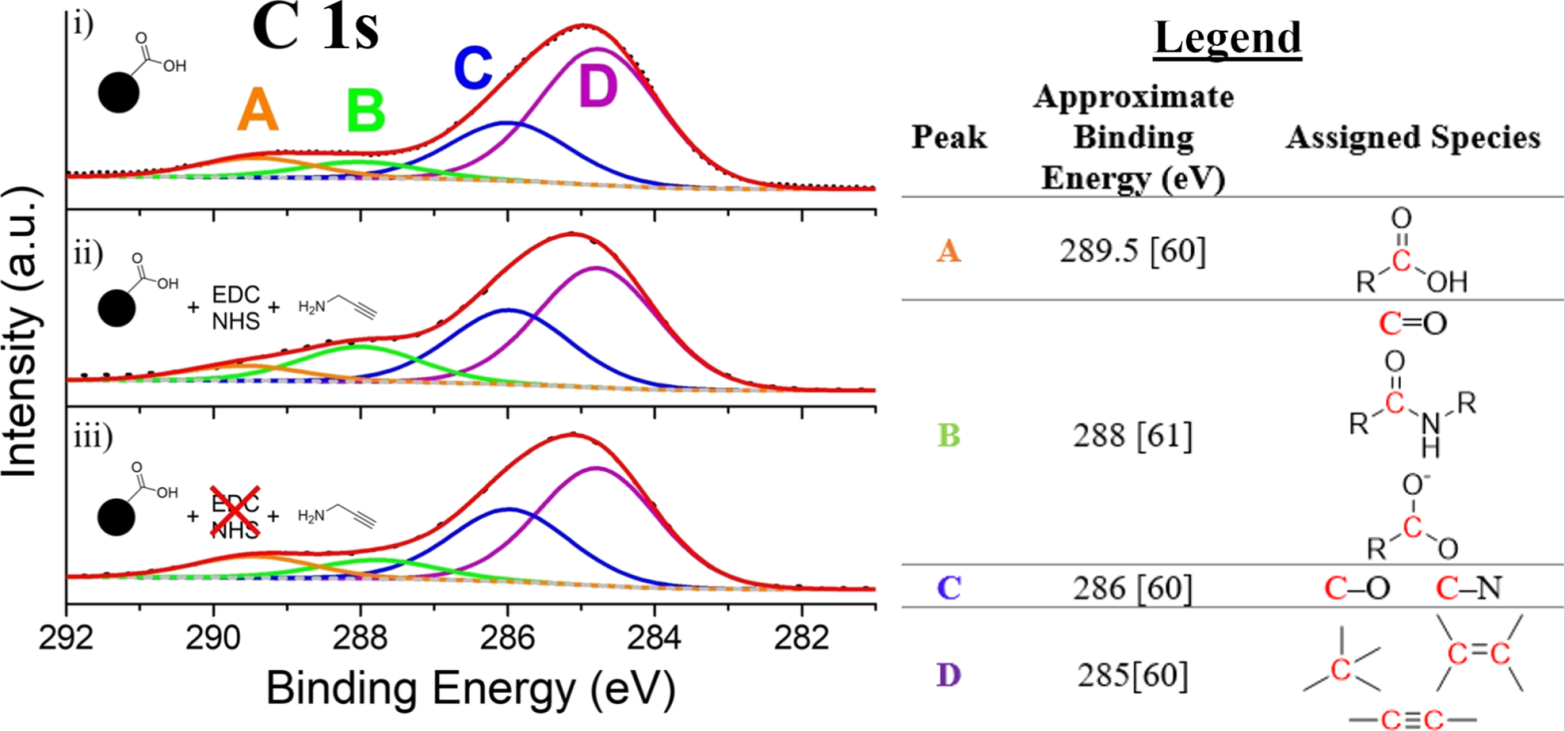
High-resolution C 1s XPS spectra of i) IONP-3,4-DHBA, ii) IONP-PPA, and iii) EDC-free PPA control. Refer to legend for assignments of peaks A–D.

The C 1s XPS spectra of IONP-3,4-DHBA, IONP-PPA, and the EDC/NHS-free PPA control are presented in Figure 4, fitted using four peaks (A–D), whose assignments can be found in the legend. Peak B is observed in all samples; however, its relative intensity is higher in IONP-PPA (Figure 4.ii). This peak is attributed to a combination of three possible species, as noted in Figure 4 (legend): (1) C=O from the quinone form of 3,4-DHBA (Figure 1a) on the IONP surface, (2) COO^−^ of 3,4-DHBA (or its quinone form), and (3) N–(C=O)- from the amide bond formed through EDC coupling on IONP-PPA [60]. Both (1) and (2) are expected for IONP-PPA and the EDC-free PPA control; however, (3) should only be present for IONP-PPA. The added contribution of the amide group to peak B in the spectrum of IONP-PPA results in the increase in the relative intensity of this component peak. We further observe that peak A (COOH) [61] has a lower relative intensity in the IONP-PPA spectrum, which is consistent with the conversion of carboxylate groups into amides through the EDC coupling reaction (i.e., peak intensity is shifted from peak A to peak B, following EDC coupling of PPA). As the EDC-free PPA control (Figure 4.iii) cannot form amide bonds in the absence of EDC activation, we see a lower relative intensity of peak B, which has contributions from (1) and (2) only, as well as a more relatively intense peak A. We further note that peaks A and B are very similar between IONP-3,4-DHBA (Figure 4.i) and the EDC-free PPA control, indicating no involvement of the carboxylate groups in the binding of PPA. This reinforces the above analysis of the FTIR spectrum of IONP-PPA, which suggested the presence of amide bonds on the IONP-PPA surface, indicating at least some PPA is binding through the intended route. Peaks C and D appear consistent, in terms of relative intensities, between all samples.

The N 1s spectra (Supporting Information File 1, Figure S2) are complicated by the closely spaced bands associated with primary amines (R–**N**H_2_), amides (**N–**(C=O)-), and surface Fe-coordinated amines (Fe–**N**H_2_), all occurring at ≈400 eV [62–63], which cannot be fitted with any meaningful accuracy. We thus cannot conclusively state whether or not PPA binds directly to surface Fe, from the XPS evidence.

**Figure 5:**
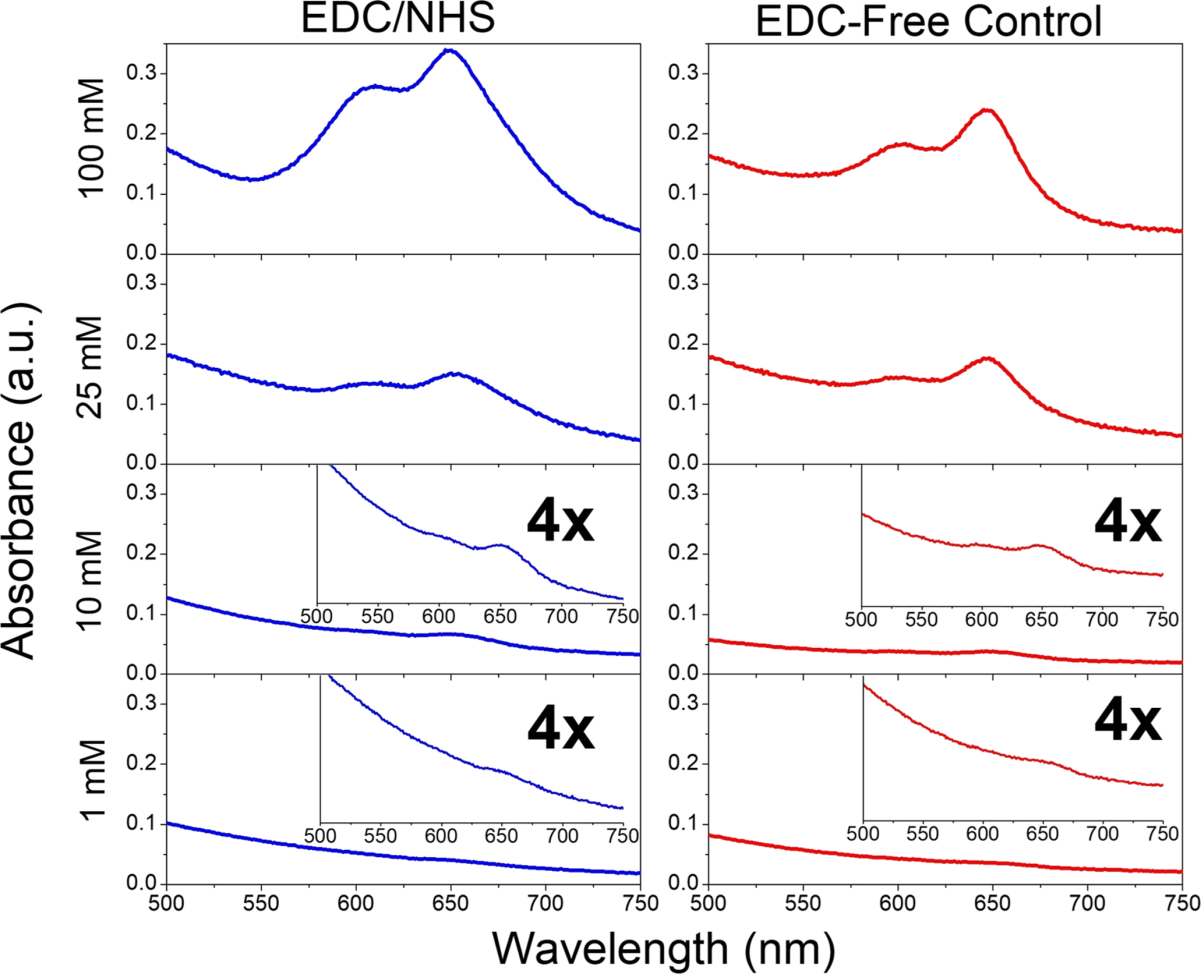
Amine concentration study of EDC/NHS-activated (left column), and EDC-free controls (right column), following CuAAC reaction with Cy5-azide, at different concentrations of PPA (1–100 mM). Insets: 4× magnified views of the respective spectra.

It should be noted that, in these experiments, PPA was added to a final concentration of 180 mM, which falls in the upper region of the typical amine concentrations used in the literature (≈0.1–300 mM) [7,13]. To demonstrate that our results are not simply an artifact of the higher amine concentration used, we performed a set of experiments using PPA of varying concentrations (1–100 mM), both in the presence and absence of EDC/NHS activation, with subsequent Cy5-azide coupling. These results are presented in Figure 5 and show that Cy5-azide coupling can be detected in both the EDC/NHS coupling products and controls, all the way down to 1 mM amine. Furthermore, at all concentrations tested, the loading of Cy5-azide is comparable for the EDC/NHS products and controls, demonstrating that the above results are not simply the result of a higher amine concentration.

In summary, the FTIR evidence suggests some amide bond formation with EDC activation, while no amides were detected in the EDC-free control, as expected. Our UV–vis results suggest a denser dye loading on IONP-PPA than the EDC-free control, which suggests more available binding sites (alkynes) on IONP-PPA. The C 1s XPS spectra also suggest the presence of amide bonds in IONP-PPA, but not in the EDC-free control, in agreement with the FTIR findings. The N 1s spectra, in contrast, are inconclusive for determining the interactions of the amine group with the IONPs. Together, the FTIR, UV–vis, and XPS evidence suggests successful EDC coupling of PPA to 3,4-DHBA ligands on the IONP surface for IONP-PPA, but also indicates the presence of an additional binding mode of PPA which occurs in both IONP-PPA and the EDC-free control. The nature of this additional binding mode could not be determined unambiguously from the above techniques; however, we can reasonably rule out ionic bonding between ammonium and carboxylate ions, as well as formation of tetra-substituted ring structures through reaction with quinones, using FTIR analysis. Given the previously discussed use of amine ligands on IONPs in the literature [47–48], the most reasonable candidate for this binding mode is, thus, coordination of the amine with surface Fe.

### EDC coupling attempts with primary amine dyes

**Figure 6:**
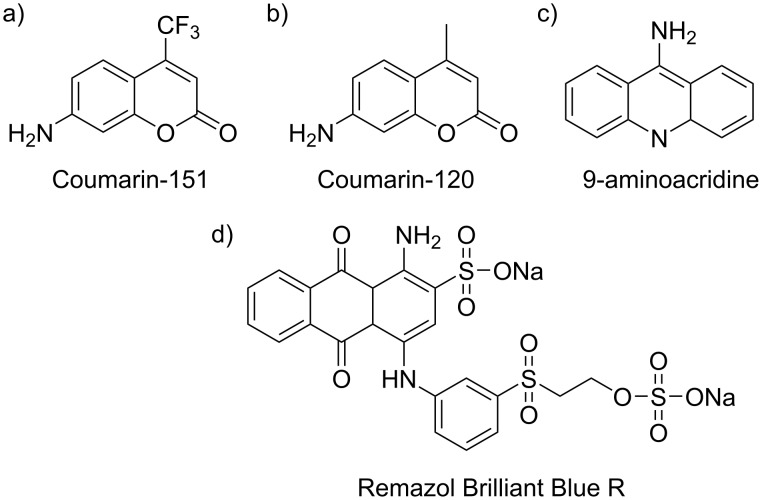
Structural formulae of the primary amine dyes used in this study.

The necessity to perform indirect methods of determining the binding of PPA makes interpretation of the EDC/NHS coupling results complicated. In an attempt to observe direct evidence of successful EDC/NHS coupling of a payload molecule to IONP-3,4-DHBA, we attempted to couple several primary amine-containing dyes. We first tried the commercially available primary amine dyes, Coumarin-151, Coumarin-120, and 9-aminoacridine (Figure 6a–c), whose presence on the IONP surface should be detectable directly by UV–vis spectroscopy. Unfortunately, we could not detect binding with any of these dyes (results not shown). All three dyes showed relatively low aqueous solubility, and have their primary amine attached directly to an aromatic ring structure, which may contribute to both steric hindrance, as well as to deactivation of the amine through withdrawal of electron density. We suspect that the latter is the most important factor affecting the poor reactivity of these dyes, as attempts to perform the EDC/NHS coupling in DMF, DMSO, and EtOH, wherein the dyes are much more soluble, also did not result in any detectable dye loading on the IONPs. We also tried using Remazol Brilliant Blue R (Figure 6d), a commercially available primary amine-containing dye with sulfonate groups, which impart excellent aqueous solubility, and once again, detected no dye on the IONPs. We thus conclude that the structure of the payload molecule plays a very important role in determining the efficacy of the EDC/NHS coupling reaction, in that steric hindrance and the electronic structure of the molecule are critical parameters to take into consideration when choosing a suitable amine; thus, efforts should be made to include a linker, which separates the primary amine from the aromatic ring structure. Importantly, we also note that there is no cross-reactivity observed here, likely due to these same factors that prevent the dyes from binding to surface Fe via their amines, unlike with PPA.

### Use of cysteamine for EDC coupling to IONPs and thiol–maleimide coupling of Cy3-maleimide

**Figure 7:**
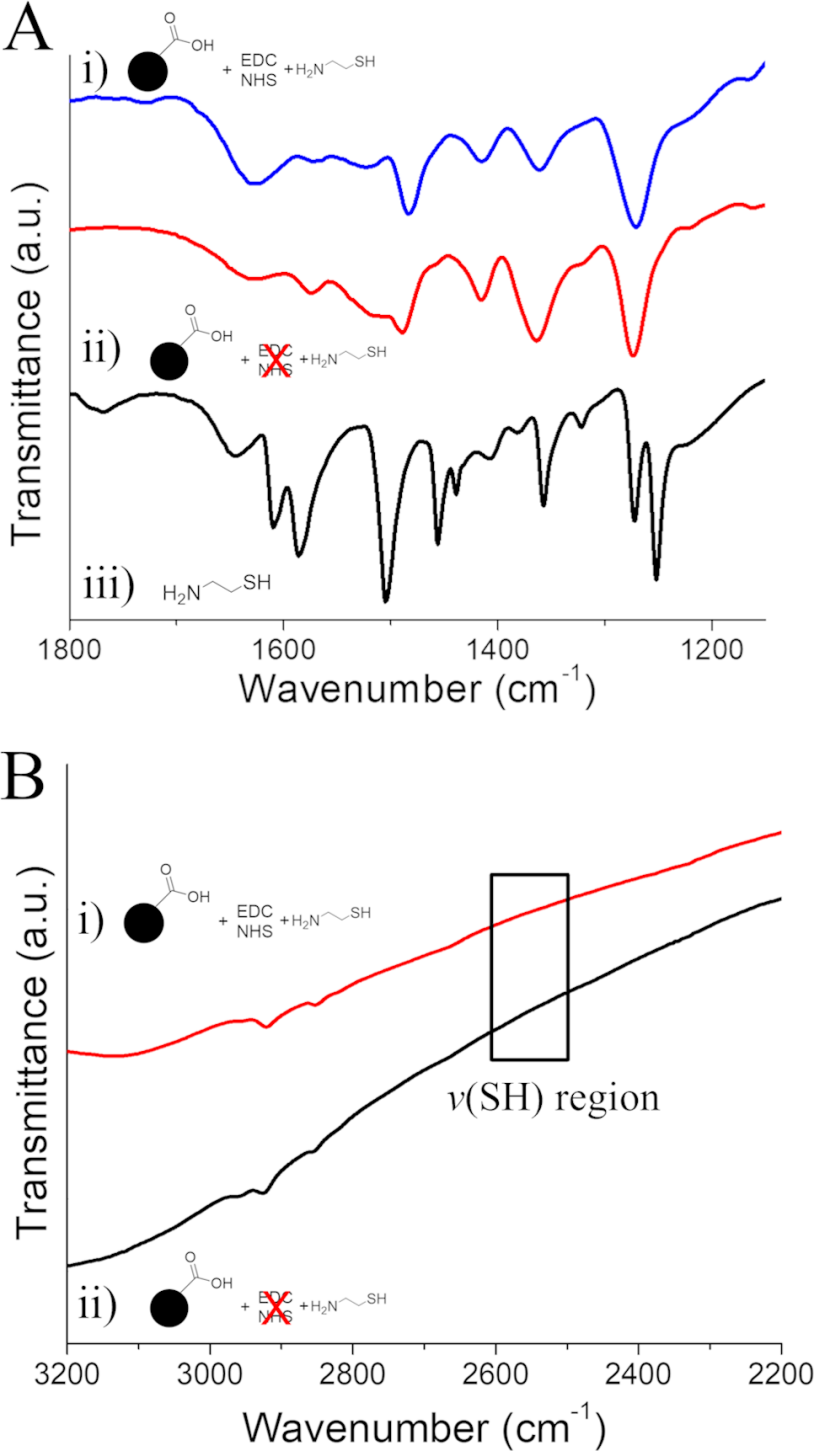
FTIR spectra of CySH products and controls. (A) the fingerprint region showing i) IONP-CySH, ii) EDC/NHS-free CySH control, and iii) pure cysteamine powder. (B) Higher-energy region of the spectrum, highlighting the SH stretch region (black box) of i) IONP-CySH and ii) the EDC/NHS-free CySH control.

To expand our investigation to other small molecule linkers with common coupling groups, we attempted to incorporate thiols onto the IONP surface through EDC/NHS coupling of CySH. The FTIR spectrum of IONP-CySH (Figure 7A.i) shows considerable similarities with that of IONP-PPA, which leads us to believe that we have successfully formed amide bonds on the IONP surface, as reasoned above. In contrast to the spectra of IONP-PPA and its control, we also observe significant differences in the bands attributed to ring stretching and bending modes on the EDC-free CySH control (Figure 7A.ii), which could possibly indicate changes to the aromatic ring structure, such as the formation of tetra-substituted rings, but this cannot be unambiguously confirmed by FTIR. We note that the FTIR spectrum of pure CySH (Figure 7A.iii) shows some intense bands that overlap with the ring modes of 3,4-DHBA, and so these may also account for the differences observed in this region of the spectrum. We also find that, in both cases, the inherently weak stretching bands of the SH group (≈2550 cm^−1^) [57] could not be observed in the FTIR spectra (Figure 7B), so an alternative means of detecting CySH on the IONP surface is necessary.

**Figure 8:**
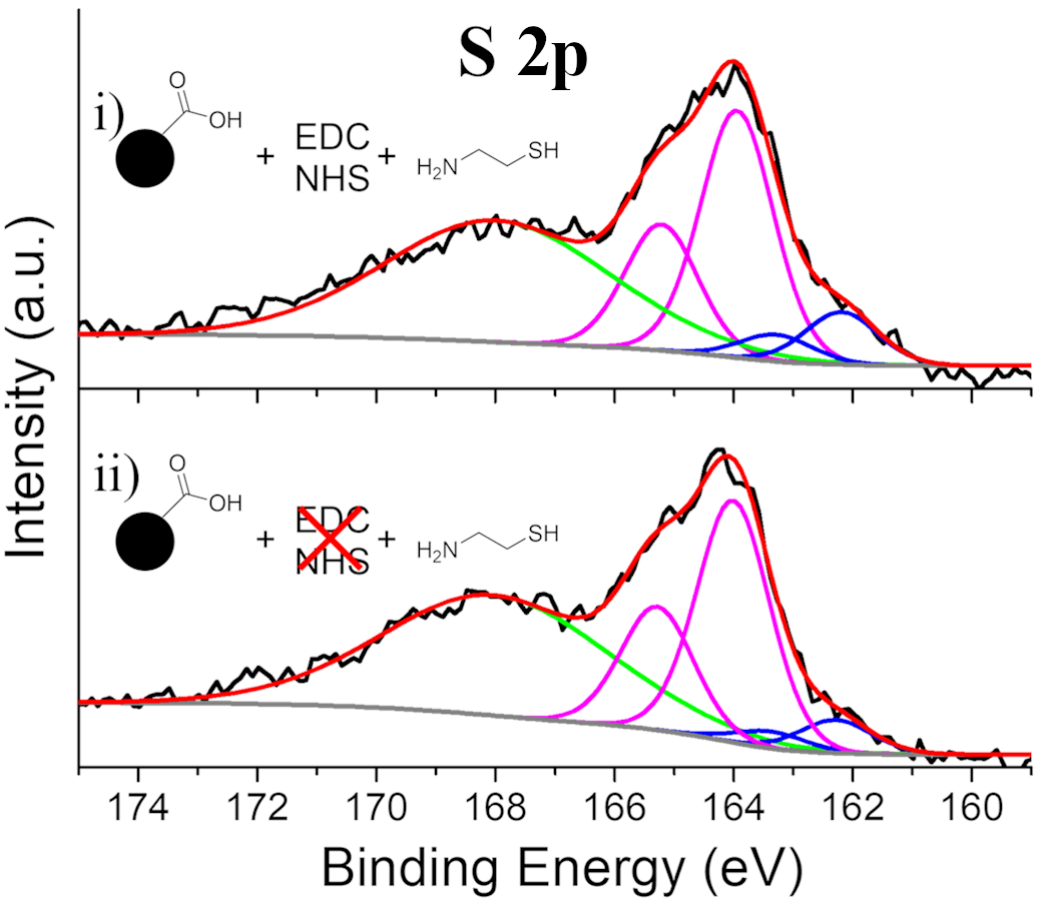
High-resolution S 2p XPS spectra of i) IONP-CySH and ii) the EDC-free CySH control.

To verify the binding of CySH, IONP-CySH and the EDC-free CySH control were analyzed by XPS. The S 2p spectrum of IONP-CySH (Figure 8.i) shows a minor amount of Fe–S content (≈18%), which suggests that the majority of the CySH is bound either to 3,4-DHBA quinones through its thiol, which is much more reactive towards quinones than the amine [58], complexation with surface Fe through its amine, or in this case, the intended EDC/NHS coupling to 3,4-DHBA. Unfortunately, resolving these contributions in the XPS spectra is not possible. The S 2p spectrum of the EDC-free CySH control (Figure 8.ii) reveals the presence of S, which is evidence of the binding of CySH in the absence of EDC/NHS activation. We also observe a minor amount of Fe–S content (≈12%), similar to IONP-CySH, which suggests that the majority of the bound CySH is either coordinated to surface Fe through the amine, or possibly bound to 3,4-DHBA quinones through its thiol. The presence of a higher binding energy feature in the S 2p spectra at >166 eV is attributed to a Si 2s plasmon loss peak, derived from the Si substrate (Supporting Information File 1, Figure S3) [64].

**Figure 9:**
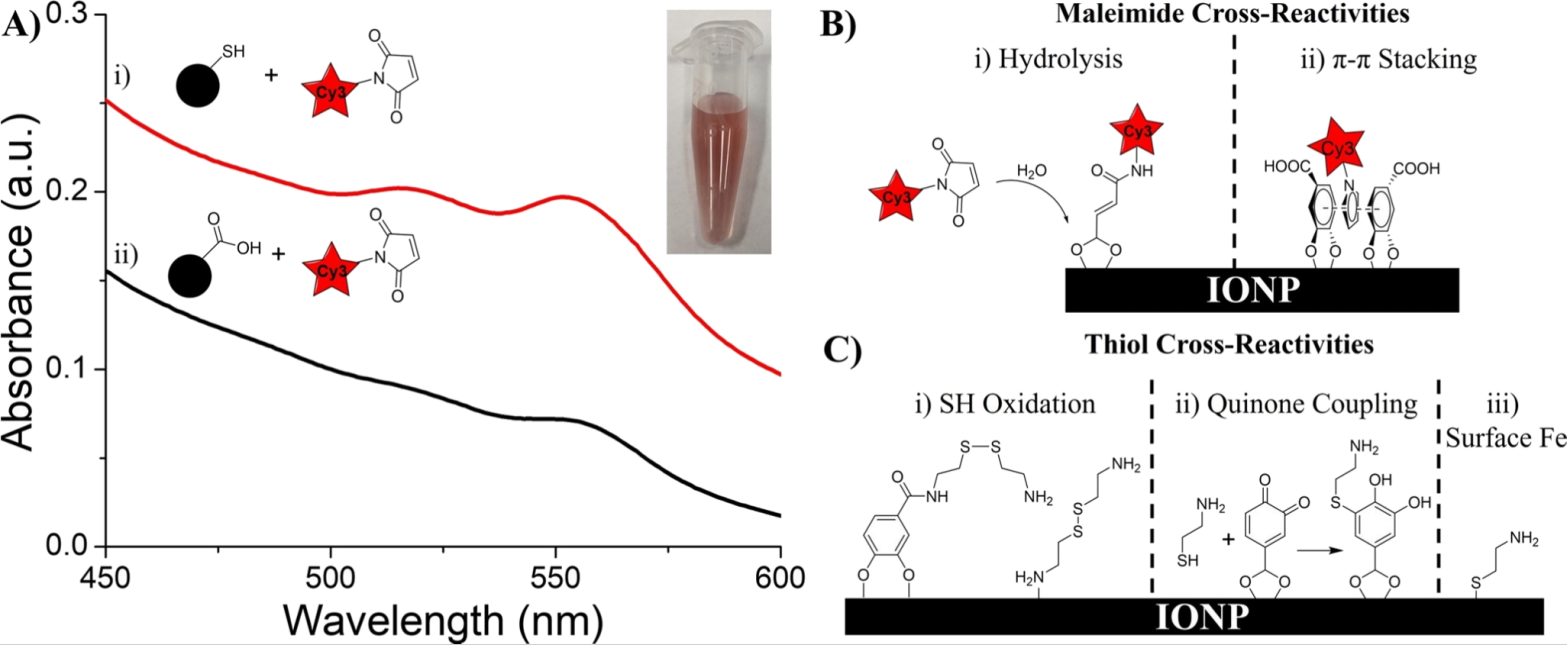
Characterization of the thiol–maleimide coupling products and controls with CySH. (A) UV–vis spectra of i) IONP-CySH product, and ii) IONP-3,4-DHBA (thiol-free) control. Inset: photograph of an aqueous suspension of thiol–maleimide coupling product of IONP-CySH. (B) Possible maleimide cross-reactivities, involving i) hydrolysis of the maleimide group to a maleic acid group, and ii) intercalation of maleimide group between 3,4-DHBA ligands, due to π–π interactions (NOTE: the carbonyl groups of the maleimide are omitted for clarity). (C) Possible thiol cross-reactions, involving i) oxidation of CySH to a disulfide, ii) reaction of the thiol with 3,4-DHBA quinones, and iii) binding of thiols directly to surface Fe.

To assess any cross-reactivity in the thiol–maleimide Michael addition reaction, we utilized Cy3-maleimide, and relied on UV–vis spectroscopy to detect the binding of Cy3. When reacted with Cy3-maleimide, the IONP-CySH product was noticeably red (Figure 9A, inset), indicating successful loading of Cy3; however, the IONP-3,4-DHBA control (no thiols) appeared pink, indicating some Cy3 loading. Sure enough, we see two peaks at ≈516 and 551 nm in the UV–vis spectrum of the IONP-CySH product (Figure 9A.i), which confirms the presence of Cy3 (Supporting Information File 1, Figure S1.ii). We also detect Cy3 on the IONP-3,4-DHBA control (Figure 9A.ii), unlike Cy5-azide, which showed no cross-reactivity with IONP-3,4-DHBA. If we consider the similar backbone structure of Cy3 and Cy5, and the fact that Cy5-azide showed no cross-reactivity with IONP-3,4-DHBA, we can reason that the cross-reactivity observed here is due to the maleimide group of Cy3-maleimide. It is possible that hydrolysis of the maleimide group under the aqueous reaction conditions forms a maleic acid group [31], allowing the Cy3 to coordinate to the IONP surface through the carboxylic acid group (Figure 9B.i). Alternatively, the possibility exists of π–π interactions between the maleimide group and the 3,4-DHBA aromatic rings, allowing it to become intercalated within the ligand layer (Figure 9B.ii).

**Figure 10:**
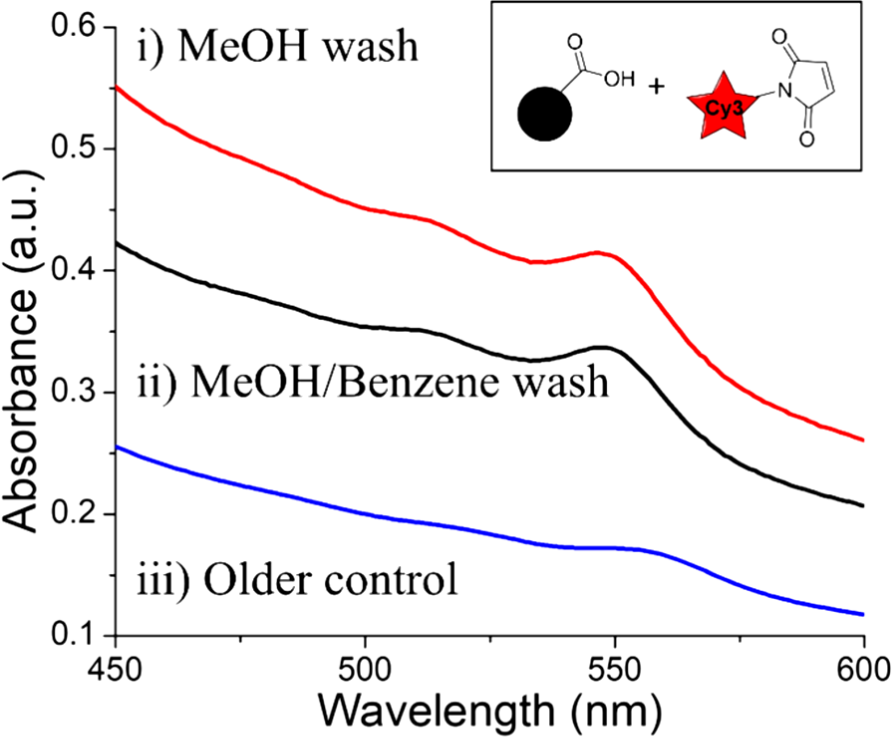
UV–vis spectra of MeOH- and MeOH/benzene-washed Cy3-maleimide treated IONP-3,4-DHBA controls. i) MeOH only wash, ii) MeOH/benzene wash, and iii) MeOH only wash from 2 years earlier, using same Cy3-maleimide stock solution (same curve as Figure 9A.ii).

To rule out the possibility of Cy3-maleimide intercalating within the ligand layer due to π–π interactions, we reacted two IONP-3,4-DHBA aliquots with Cy3-maleimide, in the absence of EDC/NHS activation and CySH, and subsequently washed the IONPs with either MeOH only, or a mixture of MeOH and ≈440 equivalents of benzene (relative to the amount of Cy3 used in the reaction). The excess benzene should disrupt the π–π interactions, thereby removing any bound Cy3-maleimide. The UV–vis spectra show no change in the Cy3 loading between the sample washed with MeOH only (Figure 10.i) and the sample washed with MeOH/benzene (Figure 10.ii), which rules out the involvement of π–π interactions in the loading of Cy3-maleimide. Furthermore, we note that these washing experiments were performed approximately two years after all other Cy3 experiments reported herein, using the same Cy3-maleimide stock solutions, which have likely hydrolyzed somewhat over this time interval. We observe that the loading of Cy3-maleimide on these later IONP-3,4-DHBA controls is significantly higher than our original IONP-3,4-DHBA control (Figure 10.iii), which suggests that the Cy3-maleimide has become more reactive towards the IONPs over time. This is consistent with our theory that the hydrolysis of the maleimide group can significantly contribute to the binding of Cy3-maleimide to the IONP surface.

The loading of Cy3-maleimide on IONP-CySH (Figure 9A.i) is observed to be much less than that of Cy5-azide on IONP-PPA (Figure 3B.i), even when the higher absorption coefficient of Cy5 (271,000 cm^−1^·M^−1^) relative to Cy3 (162,000 cm^−1^·M^−1^) is taken into consideration [65–66]. This suggests that the availability of thiols on the IONP-CySH surface is much lower than that of the alkynes on IONP-PPA. One possible explanation for this is oxidation of CySH to its disulfide form, CySS. The resulting disulfide, which is not reactive towards maleimides, can bind to the IONPs by either EDC coupling to the 3,4-DHBA ligands, or coordinating with surface Fe through its amine group (Figure 9C.i). Both of these possibilities leave only the amine, which is less reactive towards maleimides, and the disulfide, which is not reactive towards maleimides, exposed to solution. Another possibility is that the thiol group of CySH is binding to a 3,4-DHBA quinone ring structure, leaving only its amine group solution-exposed (Figure 9C.ii). Finally, as noted in the XPS analysis, some CySH binds directly to surface Fe through its thiol group, which again, leaves its less reactive amine exposed to solution (Figure 9C.iii). One or all of these possibilities may be at play, resulting in the relatively poor loading of Cy3-maleimide that we observe.

It should also be noted that, following the reaction of CySH with IONP-3,4-DHBA, in the absence of EDC/NHS activation, the reaction supernatant had a violet color (Supporting Information File 1, Figure S4), which indicates that surface Fe is being etched by CySH, resulting in a colored complex in solution. This is evidence that CySH interacts directly with the IONP surface. The exact nature of this complex is not known at this time, but it does not match the spectrum of Fe^3+^–DHBA complexes [67], which further suggests some involvement of the CySH ligands. Although only the control supernatant appeared visibly violet, the UV–vis spectrum of the reaction supernatant of IONP-CySH (Supporting Information File 1, Figure S4), shows that this complex is still present, but in smaller quantities.

It is clear that the use of CySH as a linker is complicated by potential cross-reactivities with both its amine and thiol groups, which cannot be unambiguously resolved. The FTIR spectra indicate some successful amide bond formation in EDC/NHS-activated samples, but also show significant differences in the DHBA ring stretching and bending modes in IONP-CySH and the EDC-free control. FTIR cannot, however, provide conclusive evidence about the nature of any alternative binding modes of CySH. The XPS spectra provide evidence for the binding of CySH to both IONP-CySH and the EDC-free control and suggest a minor amount of Fe–S binding in both cases. The binding of the remaining CySH, however, cannot be conclusively assigned, and may result from complexation of the amines with surface Fe, binding of thiols to 3,4-DHBA quinones, and/or EDC/NHS coupling to 3,4-DHBA. The etching of surface Fe in the EDC-free control reaction also adds another layer of complexity to the use of CySH as a small molecule linker on IONPs.

### Use of disulfide reducing agents: dithiothreitol

As an alternative route to thiol-functionalized IONPs, circumventing the potential cross-reactivities associated with the thiol group of CySH, we used an approach of first coupling a disulfide molecule, CySS, which could subsequently be cleaved to produce solution-exposed thiols. The amine groups of CySS can be EDC/NHS coupled to IONP-3,4-DHBA, while the disulfide groups have not been shown to be reactive towards surface Fe. Following conjugation to 3,4-DHBA ligands, the disulfide bonds can subsequently be cleaved using a suitable reducing agent, such as DTT or TCEP, to produce solution-exposed thiol groups.

**Figure 11:**
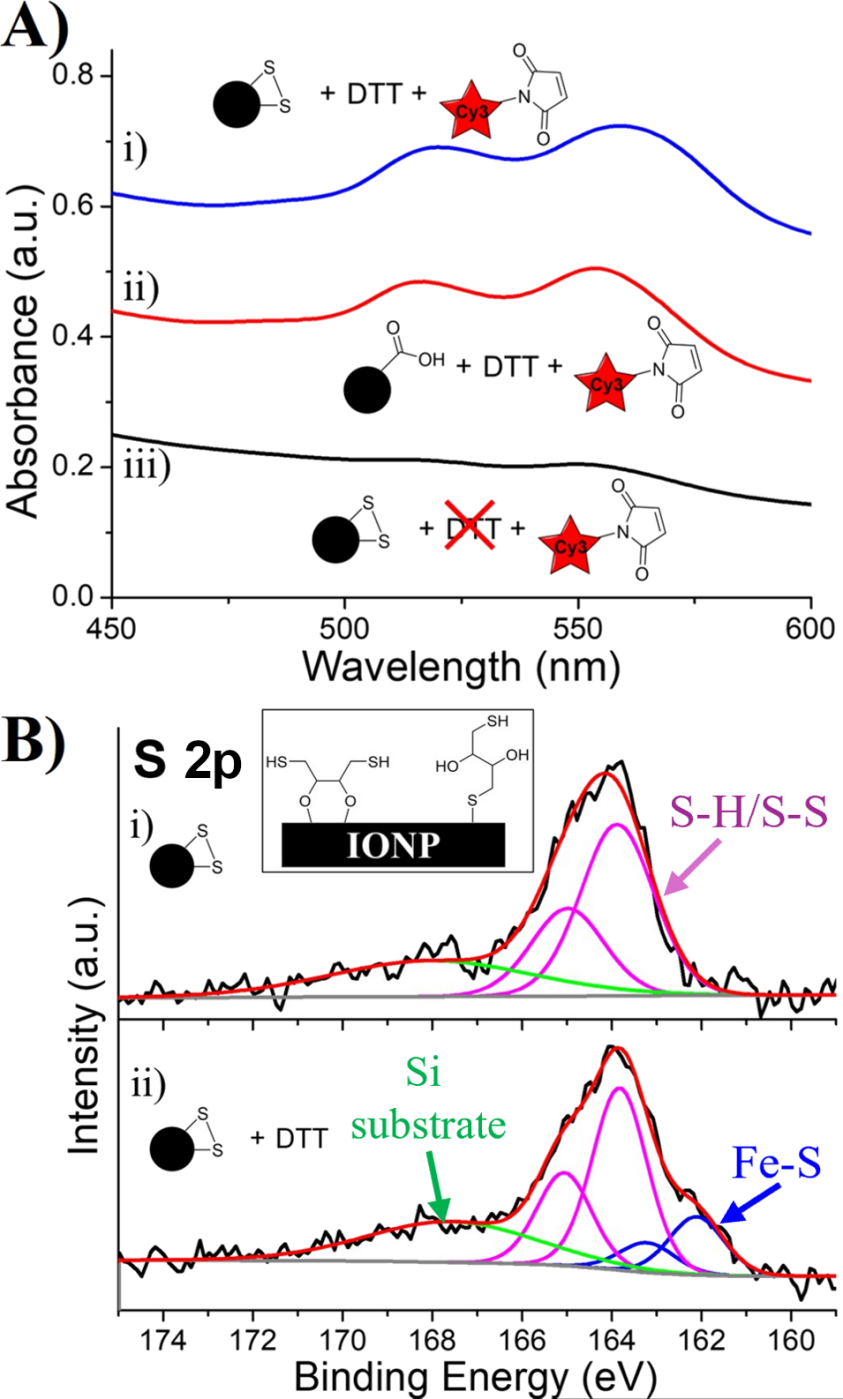
UV–Vis and XPS characterization of DTT-treated IONPs. (A) UV–vis spectra of the thiol–maleimide coupling products of i) IONP-CySS treated with DTT (IONP-CySS/DTT), ii) IONP-3,4-DHBA (no disulfides) control treated with DTT (IONP-3,4-DHBA/DTT), and iii) IONP-CySS. (B) High-resolution S 2p spectra of i) IONP-CySS, and ii) IONP-CySS/DTT. Inset: possible binding schemes of DTT on the IONP surface.

IONP-CySS was prepared by EDC/NHS-coupling CySS to IONP-3,4-DHBA, in the same manner as CySH. Following a control reaction with Cy3-maleimide, the UV–vis spectrum of IONP-CySS (Figure 11A.iii) shows very low Cy3 loading, as we would expect due to disulfides being unreactive towards maleimides. IONP-CySS was subsequently treated with DTT (Figure 1f) to cleave the disulfide bonds and produce solution-exposed thiols. Excess DTT was purified away, and the product was then reacted with Cy3-maleimide. The DTT treatment resulted in a tremendous increase in Cy3-maleimide loading (Figure 11A.i), much higher than that observed with IONP-CySH (Figure 9A.i). A control reaction was conducted, wherein IONP-3,4-DHBA (no disulfides) was treated with DTT, purified, then subsequently mixed with Cy3-maleimide, and this control showed similarly high Cy3-maleimide loading (Figure 11A.ii). Clearly, the DTT is binding to the IONPs, which then increases the surface SH content, thereby increasing the Cy3-maleimide loading significantly. This further indicates that DTT is not coordinated to the IONP surface through both of its thiol groups, but rather, binds through either the hydroxy groups, or a single SH group, leaving the other SH free to react with maleimides (Figure 11B, inset).

Similar to our observations of Cy5-azide loading, we see a difference in the lineshapes of the UV–vis spectra of the IONP-CySS/DTT product and the IONP-3,4-DHBA/DTT control, which suggests differences in the Cy3 binding modes. We observe the presence of a broadened lineshape for the IONP-CySS/DTT, which suggests the presence of multiple binding sites, resulting in an increased Cy3 packing density, thereby enabling H-aggregate formation. This observation provides evidence that there is successful cleavage of the disulfide bonds, when treated with DTT, resulting in Cy3-maleimide binding to both the cleaved CySS groups, and bound DTT ligands.

Analysis of the S 2p XPS spectrum of IONP-CySS (Figure 11B.i) reveals the absence of the low binding energy Fe–S doublet, which is expected due to the presence of disulfide bonds that cannot bind surface Fe. After treating IONP-CySS with DTT, the S 2p spectrum (Figure 11B.ii) shows the highest proportion of Fe–S contribution of all the samples (≈24%). The Fe–S bonding suggests that at least some DTT binds to the IONP surface through the SH groups, but the UV–vis results indicate that at least one of the DTT thiols must be solution-exposed to bind Cy3-maleimide. We reason that, while the DTT molecule can reach the IONP surface and bind through one of its thiols, the density of DHBA ligands is such that it is not likely that the DTT molecule will fold over and bind to surface Fe through the remaining SH group at the opposite end of the molecule as well. These results suggest that DTT makes an excellent ligand for the preparation of SH-functionalized IONP but is a poor choice of reducing agent for disulfide ligands on IONPs for this same reason. Thus, care must be taken when using DTT as a disulfide reducing agent on IONP surfaces, as any subsequent maleimide conjugation may, in fact, be due to the presence of surface-bound DTT, rather than (or in addition to) successful cleavage of the disulfide bonds.

### Use of disulfide reducing agents: tris(2-carboxyethyl)phosphine

**Figure 12:**
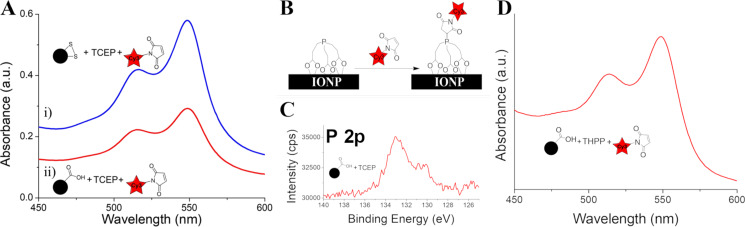
UV–vis and XPS characterization of TCEP- and THPP-treated IONPs. (A) UV–vis spectrum of thiol–maleimide coupling product of i) TCEP-treated IONP-CySS (IONP-CySS/TCEP), and ii) IONP-3,4-DHBA (no disulfides control) treated with TCEP (IONP-3,4-DHBA/TCEP). (B) Binding of TCEP to the IONP surface and reaction with Cy3-maleimide. (C) High-resolution P 2p spectrum of the IONP-3,4-DHBA control treated with TCEP. (D) UV–vis spectrum of THPP-treated IONP-3,4-DHBA control, after reaction with Cy3-maleimide.

To get around the interferences of the DTT thiols described above, we tried another commonly used disulfide reducing agent, TCEP (Figure 1g). TCEP is typically used as a disulfide reducing agent in biochemical applications, as a non-thiol-containing substitute for DTT [68]. TCEP is also more stable than DTT, which readily oxidizes at pH >7.5, and is a more effective reductant than DTT at pH <8 [69]. Treatment of IONP-CySS with TCEP resulted in a tremendous increase in Cy3-maleimide loading (Figure 12A.i), similar to that observed with DTT treatment. A control was performed wherein IONP-3,4-DHBA (no disulfides) was treated with TCEP, purified, then subsequently mixed with Cy3-maleimide. Much like with DTT treatment, this control showed very high Cy3-maleimide loading (Figure 12A.ii). It is well known that the phosphine group has a definite cross-reactivity with maleimides; thus, its presence can interfere with thiol–maleimide coupling [68,70–71]. In our case, the most likely explanation is that the carboxylate groups of TCEP are coordinating with surface iron, producing phosphine-functionalized IONPs, which then couple with Cy3-maleimide (Figure 12B), resulting in the observed increase in Cy3 loading. The IONP-3,4-DHBA/TCEP control shows less Cy3 binding than IONP-CySS/TCEP, but still more loading than IONP-CySH (Figure 9A.i). The increased Cy3 loading of IONP-CySS/TCEP, relative to the IONP-3,4-DHBA/TCEP control, may evidence successful cleavage of the disulfide bonds of CySS, resulting in contributions from both the cleaved CySS ligands and the bound TCEP to the Cy3-maleimide conjugation. We do not, however, observe any broadening in the spectra to suggest H-aggregate formation. This suggests that, despite the increased loading on the IONP-CySS/TCEP product, the Cy3 packing density is roughly the same as in the control. The binding of bulky TCEP may block some conjugated 3,4-DHBA binding sites, thereby offsetting the increased number of binding sites such that the packing density is effectively unchanged. XPS analysis of the TCEP-treated IONP-3,4-DHBA control confirms the binding of TCEP onto the IONP surface through the presence of phosphorus (Figure 12C).

We also performed a control reaction on IONP-3,4-DHBA using the TCEP analogue THPP (Figure 1h), which does not have any carboxylate groups. After reacting the product with Cy3-maleimide, we still see high Cy3 loading (Figure 12D), indicating that even THPP binds to the IONP surface. Our previous study on buffer interactions with IONPs [67] demonstrated that the buffer Tris, whose structure is analogous to THPP, showed significant binding to the IONP surface, likely through its hydroxy groups. It is clear that the addition of a disulfide cleavage step is not feasible by any conventional means due to significant cross-reactivity with the IONP surface. Most significantly, the cross-reactivities of the disulfide reducing agents all resulted in increased Cy3 loading, that, in the absence of proper controls, would suggest successful disulfide cleavage, which may only be partially correct, but does not tell the whole story.

### Interactions with IONP-3,5-DHBA

To gain further insight into the nature of the interactions between amines, thiols, maleimides and the IONP surface, we performed EDC/NHS coupling controls with PPA and CySH, using IONP-3,5-DHBA. We chose the 3,5-DHBA ligand because it has been shown to bind to the IONP surface exclusively through its carboxylate group [42], and thus should provide no means of EDC/NHS activation and amide bond formation. Furthermore, the *meta* hydroxy groups cannot form a stable quinone [56]; thus, this ligand should also eliminate the possibility of quinone cross-reactions, which may have been present with IONP-3,4-DHBA.

**Figure 13:**
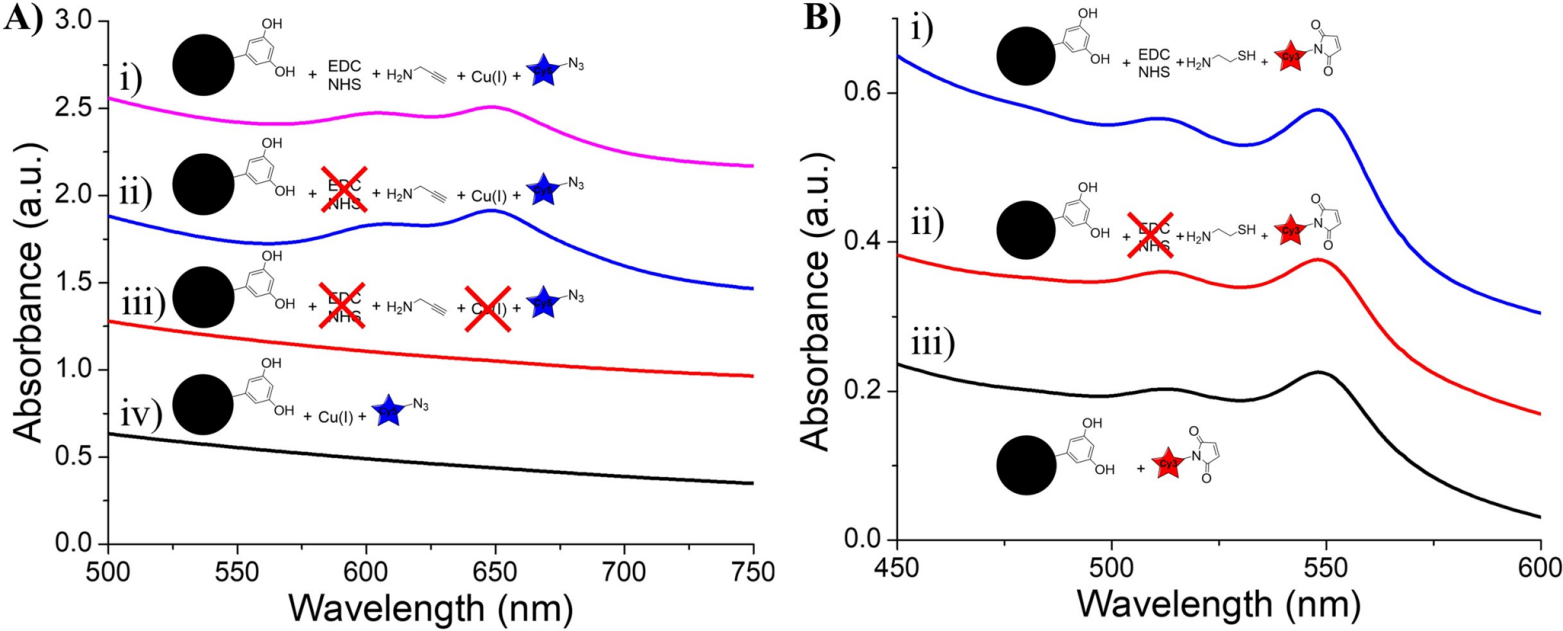
UV–vis characterization of IONP-3,5-DHBA CuAAC and thiol–maleimide coupling controls. (A) UV–vis of CuAAC reaction products of i) EDC/NHS-treated IONP-3,5-DHBA with PPA, ii) EDC-free PPA control (full CuAAC), iii) EDC-free PPA control (Cu-free), and iv) IONP-3,5-DHBA control (full CuAAC). (B) UV–vis of thiol–maleimide coupling products of i) EDC/NHS-treated IONP-3,5-DHBA with CySH, ii) EDC-free CySH control, and iii) IONP-3,5-DHBA control.

We first subjected IONP-3,5-DHBA to the EDC/NHS coupling protocol using PPA, as well as controls in the absence of EDC/NHS activation. The binding of PPA was, again, verified through the CuAAC using Cy5-azide, and controls were performed at this step to ensure that any Cy5 binding was indeed the result of the CuAAC. From the UV–vis spectra, we see that even in the absence of surface carboxylate groups, there is significant binding of PPA, as evidenced by the Cy5-azide loading on the EDC/NHS-treated (Figure 13A.i) and EDC-free (Figure 13A.ii) PPA controls, as was observed in IONP-3,4-DHBA (Figure 3C). When PPA is bound to IONP-3,5-DHBA, in the absence of EDC/NHS treatment, we observe no binding of Cy5-azide when the Cu(I) catalyst is omitted (Figure 13A.iii). Our controls demonstrate that IONP-3,5-DHBA itself does not bind Cy5-azide (Figure 13A.iv), as observed with IONP-3,4-DHBA. This provides strong evidence that the loading of Cy5-azide on the IONP surface is still the result of the CuAAC; thus, PPA must be involved and must be binding to the IONP-3,5-DHBA surface through its amine. As the formation of *meta*-quinones are highly unfavorable, it is likely that the binding of PPA to IONP-3,5-DHBA occurs not through the 3,5-DHBA ligand, but rather, through complexation with surface Fe. We further observe no difference in the broadening of the UV–vis spectra to indicate a difference in packing density of Cy5 between the EDC and EDC-free samples, which suggests the same binding sites are available in both cases, namely, surface Fe sites.

We similarly performed a set of EDC/NHS coupling controls on IONP-3,5-DHBA using CySH, with subsequent thiol–maleimide coupling of Cy3-maleimide, to probe the interactions at the IONP surface (Figure 13B). We see that, once again, there is significant binding of Cy3-maleimide, when treated with EDC/NHS (Figure 13B.i), when CySH is combined with IONP-3,5-DHBA in the absence of EDC/NHS treatment (Figure 13B.ii), and in the absence of CySH (IONP-3,5-DHBA control) (Figure 13B.iii). Furthermore, the Cy3-maleimide loading is comparable in all three samples, which suggests that CySH may have very minimal involvement in the loading of Cy3-maleimide to the IONP surface. We again consider the possibility that hydrolysis of the maleimide group into a maleic acid group allows for the Cy3 to coordinate directly to surface Fe. The similarity in cross-reactivities observed on IONP-3,4-DHBA and IONP-3,5-DHBA suggests that the IONP surface itself is the culprit in these unwanted couplings, as opposed to quinones derived from our choice of ligand. The fact that carboxylate binding is the only mode available for 3,5-DHBA to the IONP surface also rules out any ammonium–carboxylate ionic bonding.

## Conclusion

We have demonstrated significant cross-reactivity between iron oxide nanoparticles and common coupling functional groups, as well as disulfide reducing agents. We showed that PPA binds to IONPs, most likely directly to surface Fe through its amine, leaving the alkyne group free for CuAAC coupling. Without proper controls, this result could easily be misinterpreted as successful EDC coupling of PPA to carboxylated ligands, when in fact, this may only be partly true. Conversely, for the CuAAC, we find no significant cross-reactivities between alkynes or azides with the IONP surface. Although we used an amine concentration towards the higher end of the typical range for EDC coupling on IONPs, our concentration study with PPA demonstrates that our findings are applicable down to 1 mM, limited by the detection limit of Cy5. Our investigation into the EDC coupling of several primary amine-containing dyes shows that care must be taken in choosing an appropriate amine for coupling, as the structure can inhibit coupling through either steric or electronic effects.

Our results similarly demonstrate cross-reactivity of CySH with the IONP surface, possibly through both the thiol and amine groups, with surface Fe, illustrating the potential complications which can arise when using small molecule linkers with such groups. We further demonstrate extensive cross-reactivity of Cy3-maleimide with the IONPs, which may originate from binding of hydrolyzed maleimide groups directly to surface Fe. Once again, without proper controls, these results would suggest successful EDC coupling of CySH to the IONP surface, resulting in successful thiol–maleimide coupling of Cy3-maleimide.

When the disulfide ligand, CySS, is conjugated to the IONPs, attempts at cleaving the disulfide bonds with reducing agents showed significant binding of DTT, TCEP, and THPP to the IONP surface, and subsequent cross-reactivity with Cy3-maleimide. Without proper controls, these results could easily be misinterpreted as successful disulfide cleavage, leading to exceptional enhancement in thiol–maleimide coupling efficiency.

In order to rule out cross-reactivities with the 3,4-DHBA ligand, or its quinone form, we performed a set of coupling reactions and controls using IONP-3,5-DHBA, which offers no solution-exposed carboxylate groups for EDC coupling and cannot form stable quinones. The results suggest that the cross-reactivities of amines, thiols, and reducing agents observed herein are likely with surface Fe, and not with the DHBA ligands, thus demonstrating the broader applicability of these findings to other IONP preparations. A thick polymer shell may protect the IONPs from these cross-reactivities, but this also significantly increases their hydrodynamic diameter, which may not be favorable, depending on the application. Regardless, proper controls should be performed and reported to rule out the possibility of these cross-reactivities producing false positive results.

Finally, although these findings demonstrate numerous false positive results, which still indicate successful binding of the desired payload, it is important to understand that the means of loading are not through the intended routes. This may have important implications for the functionality and safety of these materials, especially in the case of biomedical applications, where this may have serious consequences on the safety of these materials inside the body, and thus, the importance of the use and reporting of proper controls cannot be understated.

## Experimental

### Materials

Hexane (Chromasolv®, ≥95%), chloroform (Chromasolv® Plus, ≥99.9%, stabilized with amylenes), dimethylsulfoxide (DMSO) (≥99.9%), 3-(*N*-morpholino)propanesulfonic acid (MOPS) (≥99.5%), MOPS sodium salt (≥99.5%), 2-(*N*-morpholino)ethanesulfonic acid (MES) (≥99%), MES sodium salt (≥99%), propargylamine (PPA) (98%), copper(II) sulfate anhydrous (≥99.99%), tris(hydroxypropyltriazolylmethyl)amine (THPTA) (95%), (+)-sodium ʟ-ascorbate (BioXtra, ≥99.0%), 1-ethyl-3-(3-dimethylaminopropyl)carbodiimide hydrochloride (EDC) (≥99.0%), *N*-hydroxysuccinimide (NHS) (98%), cysteamine (CySH) (≥98.0%), cystamine dihydrochloride (CySS) (≥98.0%), tris(2-carboxyethyl)phosphine hydrochloride (TCEP) (≥98%), tris(3-hydroxypropyl)phosphine (THPP) (≥80%), Coumarin-151 (≥99%), Courmarin-120 (99%), 9-aminoacridine (≥97.0%), and Remazol Brilliant Blue R (40–65% dye content) were purchased from Sigma-Aldrich (St. Louis, MO). 3,4-Dihydroxybenzoic acid (3,4-DHBA) (97%) was purchased from Alfa Aesar (Tewksbury, MA, USA). 3,5-Dihydroxybenzoic acid (3,5-DHBA) (>98.0%) was purchased from TCI America (Portland, OR). ᴅʟ-dithiothreitol (DTT) (≥99%) was purchased from MP Biomedicals (Solo, OH). Methanol (MeOH) (≥99.8%) were purchased from ACP Chemicals (Montreal, QC). Anhydrous ethanol (EtOH) was obtained from Commercial Alcohols (Brampton, ON). Benzene (99.00%) was purchased from Caledon Laboratories (Georgetown, ON). Sulfo-Cy3-maleimide (hereafter, referred to simply as Cy3-maleimide) (≥95%) and sulfo-Cy5-azide (hereafter, referred to simply as Cy5-azide) (95%) were purchased from Lumiprobe Corporation (Hunt Valley, MD). All sonication steps were performed using a Branson 2510 ultrasonic cleaner (Branson Ultrasonics Corp., Brookfield, CT) at room temperature (RT). All centrifugation steps were performed at 4000*g*, for 3 min, at rt.

#### Synthesis of 3,4-DHBA exchanged nanoparticles (IONP-3,4-DHBA)

Oleic acid-capped iron oxide nanoparticles (IONP-OA) were prepared in accordance with a previously established method, described in more detail elsewhere [50,72]. Ligand exchange was carried out based on a modified version of the methodology established in [42]*.* Briefly, 3,4-DHBA (40.7 mg, 0.26 mmol) was dissolved in 1 mL of methanol and added to 5 mg of IONP-OA in 14 mL of chloroform. The reaction mixture was sonicated for 0.5 h at rt. The resulting nanoparticles, which were no longer stable in chloroform, were collected by centrifugation and washed once with methanol. This same protocol is used to produce IONP-3,5-DHBA.

#### Coupling of amine linkers to NHS-activated nanoparticles (IONP-PPA, IONP-CySH, and IONP-CySS)

Here, we describe the procedure for preparing IONP-PPA, however, the same protocol is used for preparing IONP-CySH and IONP-CySS by the coupling of CySH and CySS, respectively. A solution of 21.4 mg (82 µmol) NHS in 0.250 mL 0.1 M MES (pH 5) was added to 0.3 mg (by total Fe) of IONP-3,4-DHBA and sonicated for 10 s to resuspend. A solution of EDC (14.5 mg, 76 µmol) in 0.250 mL of MES was added to the IONP solution, and the mixture allowed to activate in the dark for 30 min. The IONP-NHS product was pelleted by centrifugation, and the supernatants removed.

IONP-NHS was resuspended in a solution of 0.35 µL PPA (5.5 μmol) in 29.6 µL of 0.1 M pH 7.8 MOPS with brief sonication. MOPS buffer was used to prevent interferences from amine- or carboxylate-containing buffers, as well as possible cross-reactivities with the IONP surface, as we have previously demonstrated [67]. The mixture reacted in the dark at rt for 2 h. The product was pelleted by centrifugation and washed with MeOH twice before subsequent click reactions.

#### Copper-catalyzed azide-alkyne cycloaddition (CuAAC) of Cy5-azide onto alkyne-functionalized nanoparticles

The entire purified IONP-PPA pellet from above was resuspended in 31.8 µL of 0.1 M MOPS (pH 7.8) and 2 μL of 20 mM Cy5-azide (in EtOH) was added. A solution of Cu-THPTA complex was prepared by combining 2.5 μL of 0.2 M CuSO_4_ with 7.15 μL of 70 mM THPTA, and 1.54 µL of this solution was added to the reaction mixture. Finally, 4.64 μL of freshly prepared 50 mM sodium ascorbate in MOPS was added to start the reaction. The reaction proceeded for 2 h at rt in the dark. The products were pelleted by centrifugation, and the recovered nanoparticles were washed twice with methanol, and then resuspended in 1.0 mL of 0.1 M MOPS (pH 7.8) for UV–visible spectroscopy.

To demonstrate that the binding of Cy5-azide to the IONP-PPA nanoparticles is indeed the result of the CuAAC, a control of the above reaction was performed without the addition of Cu-THPTA complex. Furthermore, to show the importance of the presence of a terminal alkyne on the surface of the nanoparticles for the binding of Cy5-azide to occur, a separate control reaction was performed as above, using IONP-3,4-DHBA in place of IONP-PPA.

#### Thiol–maleimide Michael addition of Cy3-maleimide onto thiol-functionalized nanoparticles

The entire purified IONP-CySH pellet from above was resuspended in 33.6 μL of 0.1 M MOPS (pH 7.8), and a solution of 6.40 μL of 20 mM Cy3-maleimide (in DMSO) was added. The mixture was allowed to react at rt for 2 h in the dark. After the reaction was complete, the product was collected by centrifugation and washed three times with methanol. Purified IONP pellets were resuspended in 1 mL of 0.1 M MOPS (pH 7.8) for UV–visible spectroscopy.

To verify that the binding of Cy3-maleimide is the result of the thiol–maleimide coupling reaction, a control of the above reaction was performed using IONP-3,4-DHBA in place of IONP-CySH.

#### Disulfide cleavage using DTT

The entire purified pellet of IONP-CySS from above was resuspended in 25.0 μL of freshly prepared 75 mM DTT solution in 0.1 MOPS (pH 7.8) with brief sonication and allowed to react in the dark for 15 min at rt. IONPs were then collected by centrifugation and washed twice with MeOH for further use or analysis.

#### Disulfide cleavage using TCEP

A solution of 50 mM TCEP was freshly prepared in 0.1 M MOPS (pH 7.8). The entire purified pellet of IONP-CySS from above was then resuspended in 25.0 μL of 50 mM TCEP solution with brief sonication and allowed to react in the dark for 10 min. The IONPs were then collected by centrifugation and washed twice with MeOH for further use or analysis.

#### Characterization

All absorption measurements were performed on a Cary 100 Bio UV–visible spectrophotometer using Cary WinUV software v.3.00 (Agilent Technologies Inc., Santa Clara, CA, USA). The as-prepared Cy5- and Cy3-modified nanoparticle solutions in pH 7.8 MOPS, as well as the as-prepared controls, were used to collect absorption spectra. All UV–vis spectra presented in this work are vertically offset with respect to each other for clarity. The spectra are also vertically offset arbitrarily, such that the minimum absorbance within each relevant spectral window corresponds to 0 absorption units, which allows for easier comparison of peak absorptions between different figures. Within a given figure, all spectra are presented at the same scale. All IONP suspensions were prepared to approximately the same concentration for UV–vis analysis; however, some error can be expected due to small losses during purification and differences in colloidal stability between samples, so some caution must be used in making direct comparisons of absorption intensity.

ATR-FTIR spectra were collected using a Spectrum Two FTIR spectrophotometer equipped with a diamond ATR accessory and processed using Spectrum FTIR software (PerkinElmer Inc., Waltham, MA, USA). All spectra were recorded between 4000 and 400 cm^−1^, with 4 cm^−1^ resolution, averaged over 64 scans. For FTIR analysis, a slurry was prepared in methanol using an aliquot of purified nanoparticles. The prepared FTIR aliquots of IONP slurries were applied to the ATR crystal and allowed to air dry in between applications, until a sufficiently thick film was produced for spectrum acquisition.

XPS measurements were performed on a Thermo Scientific K-Alpha X-ray photoelectron spectrometer, using a monochromated Al Kα source, and the data was collected and processed using Thermo Avantage © software (v5.9912) and OriginPro 8.5. The X-ray spot size was 400 μm, and the flood gun was utilized to reduce sample charging. All samples were charge shift corrected to the C 1s peak (284.8 eV). Survey scans were collected at 200 eV pass energy, and 50 ms dwell time. High-resolution scans were collected at 150 eV pass energy, and 50 ms dwell time. A slurry of IONP sample in MeOH was applied in portions to a Si wafer and dried by blowing N_2_ gently across the surface at rt in between applications, until a uniform IONP film was obtained. The Si wafers were then stored overnight at RT, under vacuum, to thoroughly dry prior to XPS analysis.

## Supporting Information

File 1 UV–vis of pure cyanine dyes, N 1s XPS spectrum of IONP-PPA, S 2p XPS spectrum of Si substrate, UV–vis of supernatant from CySH reaction and control.

## Data Availability

Data generated and analyzed during this study will be openly available in Figshare at https://doi.org/10.6084/m9.figshare.29124638 following an embargo period of 6 months from the date of submission.
