# Supplementary material for: Cross-reactivities in conjugation reactions involving iron oxide nanoparticles
Source: Beilstein J Nanotechnol. 2025 Aug 29;16:1504–21. doi: 10.3762/bjnano.16.106 (PMC12415915; doi:10.3762/bjnano.16.106)
Supplement: File 1 — UV–vis of pure cyanine dyes, N 1s XPS spectrum of IONP-PPA, S 2p XPS spectrum of Si substrate, UV–vis of supernatant from CySH reaction and control. [file Beilstein_J_Nanotechnol-16-1504-s001.pdf]

BEILSTEIN JOURNAL OF NANOTECHNOLOGY

Supporting Information

for

Cross-reactivities in conjugation reactions involving iron oxide nanoparticles

Shoronia N. Cross, Katalin V. Korpany, Hanine Zakaria and Amy Szuchmacher Blum

Beilstein J. Nanotechnol. **2025,** *16,* 1504–1521. doi:10.3762/bjnano.16.106

UV–vis of pure cyanine dyes, N 1s XPS spectrum of IONP-PPA, S 2p XPS spectrum of Si substrate, UV–vis of supernatant from CySH reaction and control

License and Terms: This is a supporting information file under the terms of the Creative Commons Attribution License (https://creativecommons.org/licenses/by/4.0). Please note that the reuse, redistribution and reproduction in particular requires that the author(s) and source are credited and that individual graphics may be subject to special legal provisions.
The license is subject to the *Beilstein Journal of Nanotechnology* terms and conditions: (https://www.beilstein-journals.org/bjnano/terms)

Supporting Data	Number	Page
UV–vis spectra of aqueous solutions of the cyanine dyes	Figure S1	2
High resolution N 1s spectrum of IONP-PPA.	Figure S2	2
XPS spectra of the bare Si wafer substrate	Figure S3	3
UV–vis of reaction supernatant from CySH reaction and control	Figure S4	3

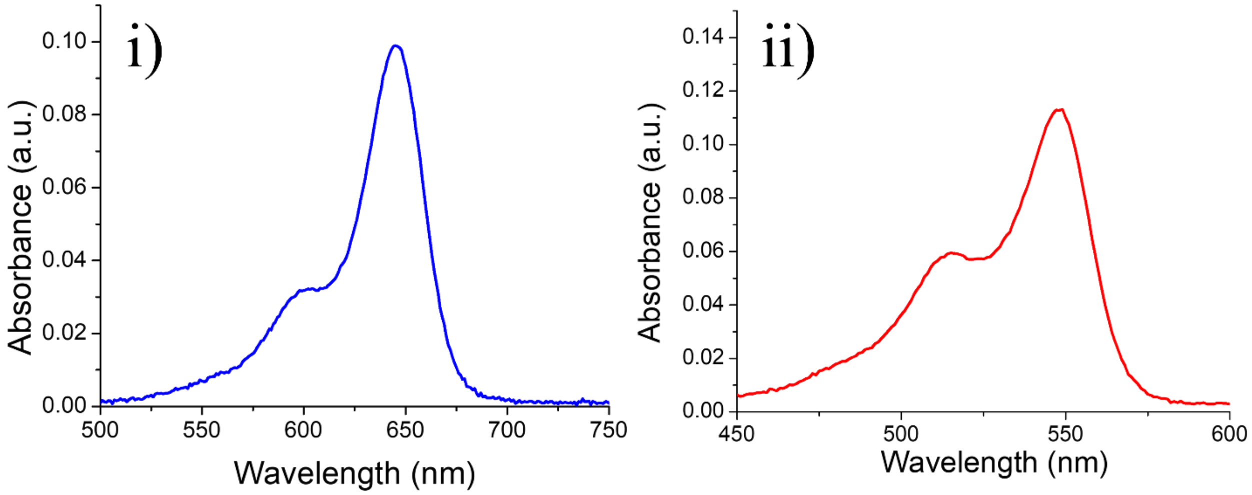

Figure S1: UV–vis spectra of aqueous solutions of the cyanine dyes used in this work. i) 1 µM Sulfo-Cy5-azide in 30 mM MOPS (pH 7), and ii) 2 µM sulfo-Cy3-maleimide in 30 mM MOPS (pH 7).

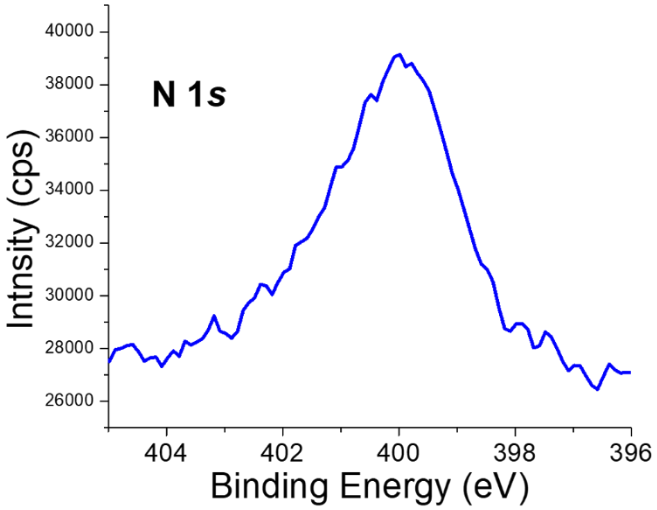

Figure S2: High resolution N 1s spectrum of IONP-PPA.

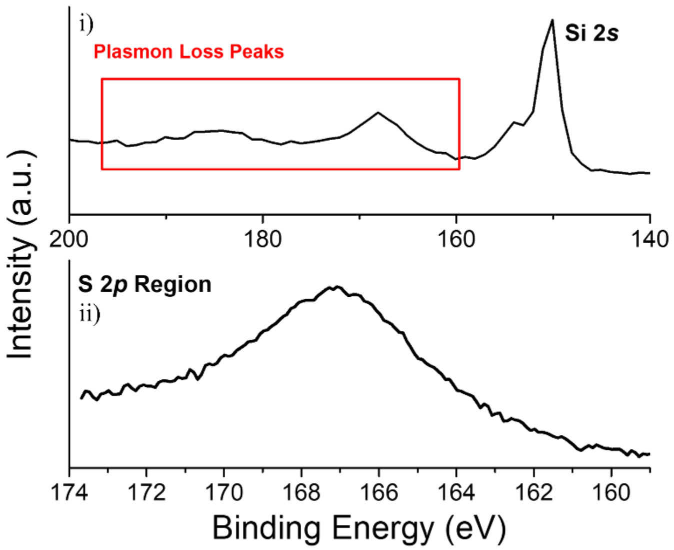

Figure S3: XPS spectra of the bare Si wafer substrate. i) Section of a survey spectrum illustrating the plasmon loss peaks from the Si 2s peak (red box), and ii) the high-resolution S 2p region, illustrating the broad Si 2s plasmon loss peak of the Si wafer substrate.

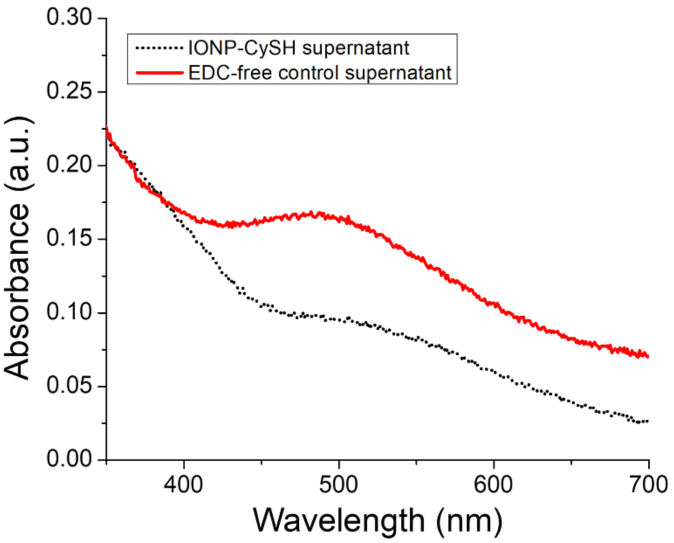

Figure S4: UV–vis spectrum of the reaction supernatant produced following reaction of IONP-3,4-DHBA with CySH. Red solid curve: reaction supernatant in the absence of EDC/NHS activation; black dotted curve: IONP-CySH reaction supernatant. λ_{max} = 485 nm.
